# Comparison of large single and small multiple doses of cyclophosphamide exposure in mice during early prepubertal age on fertility outcome

**DOI:** 10.1038/s41598-024-82264-3

**Published:** 2024-12-28

**Authors:** Sujith Raj Salian, Akshatha Daddangadi, Dhakshanya Predheepan, Divya Deeleep Bhagat Amonkar, Riddhi Kirit Pandya, Sindhura Lakshmi Koulmane Laxminarayana, Shubhashree Uppangala, Guruprasad Kalthur, Richard A. Anderson, Satish Kumar Adiga

**Affiliations:** 1 Centre of Excellence in Clinical Embryology, Department of Reproductive Science, Kasturba Medical College, Manipal. Manipal Academy of Higher Education, Manipal, 576 104, India; 2 Department of Pathology, Kasturba Medical College, Manipal. Manipal Academy of Higher Education, Manipal, 576104 India; 3 Division of Reproductive Genetics, Department of Reproductive Science, Kasturba Medical College, Manipal. Manipal Academy of Higher Education, Manipal, India; 4 Division of Reproductive Biology, Department of Reproductive Science, Kasturba Medical College, Manipal. Manipal Academy of Higher Education, Manipal, India; 5 Centre for Reproductive Health, University of Edinburgh, Edinburgh, UK

**Keywords:** Cyclophosphamide, Female infertility, Fertility preservation, Ovarian follicle reserve, Ovulation, Postnatal mortality, Infertility, Gonadal disorders

## Abstract

Cyclophosphamide (CY) exposure is known to affect the ovary and impair fertility. Clinically, treatment is generally given over multiple doses, but research models have generally used single doses. The relative effects of administering multiple small doses of CY in the prepubertal period are not elucidated. Two-week-old early-prepubertal Swiss albino female mice were administered with either large single (200 mg/Kg x 1; CY200X1) or small multiple (75 mg/Kg x 4; CY75X4) CY doses, thus a 50% higher total dose. Surviving females were assessed for estrous cyclicity, ovarian follicle reserve, oocyte functional competence, and postnatal assessment of first-generation (F1) pups. Exposure to CY75X4 reduced the loss of ovarian follicles (*p* < 0.05), and body weight (*p* < 0.001), and resulted in a larger population of cycling females (*p* < 0.01) with higher oocyte yield (*p* < 0.05) compared to CY200X1. Although CY200X1 exposed cycling females had comparable oocyte quality, and fertility index, the postnatal mortality was higher in F1 pups (*p* < 0.05) in comparison to the CY75X4 group. Although both strategies affect oocyte quality and functional competence similarly, CY75X4, despite the higher overall dose, results in reduced follicle loss, produces higher oocyte/blastocyst yield, and exhibits lower postnatal mortality rates, suggesting a potential advantage over CY200X1 for later fertility and offspring health. The differences in effects of the two treatment models show the need for designing animal model studies that more closely mimic the clinical administration of gonadotoxic therapies such as cyclophosphamide.

**Supplementary Information:**

The online version contains supplementary material available at 10.1038/s41598-024-82264-3.

## Introduction

Cyclophosphamide (CY) is a commonly used chemotherapeutic agent in pediatric oncology and in certain autoimmune diseases^1,2^. CY targets both growing and resting follicles in adult ovaries   ^3^, whereas, in prepubertal ovaries, CY directly acts on quiescent primordial follicles^4,5^. The deleterious effects of CY on prepubertal ovarian reserve are dose-dependent^6^, and a large single dose was more detrimental than a small single dose of CY to primordial follicles^4^. Our previous observation suggested that the ovaries of two-week-old mice have increased susceptibility to single doses of 200 mg/Kg CY compared to three- and four-week-old mice^7^. Conception is challenging post-CY exposure and involves risks related to fetal anomalies in mice^8^. Therefore, it is imperative to consider adapting strategies that reduce ovarian toxicity.

Impairments in oocyte and granulosa cells were found in the surviving follicles of the depleted ovarian reserve, possibly as a long-term consequence of CY exposure in adult mice^9^. Administration of small multiple doses of chemo-drugs has been studied as an alternative to the conventional regimen of chemotherapy and is associated with reduced follicle loss^4^ and reduced side effects^10,11^. Due to limited knowledge of the fertility outcome of the small multiple doses of CY exposure in prepubertal life, this study was conducted in a mouse model where small multiple (75 mg/Kg x 4) and large single (200 mg/Kg) CY doses were administered to understand their impact on ovarian reserve, oocyte competence, embryo quality, and pregnancy outcomes.

## Results

### CY exposure differentially affects body weight, survival rates, and ovarian cyclicity

**Fig. 1:**
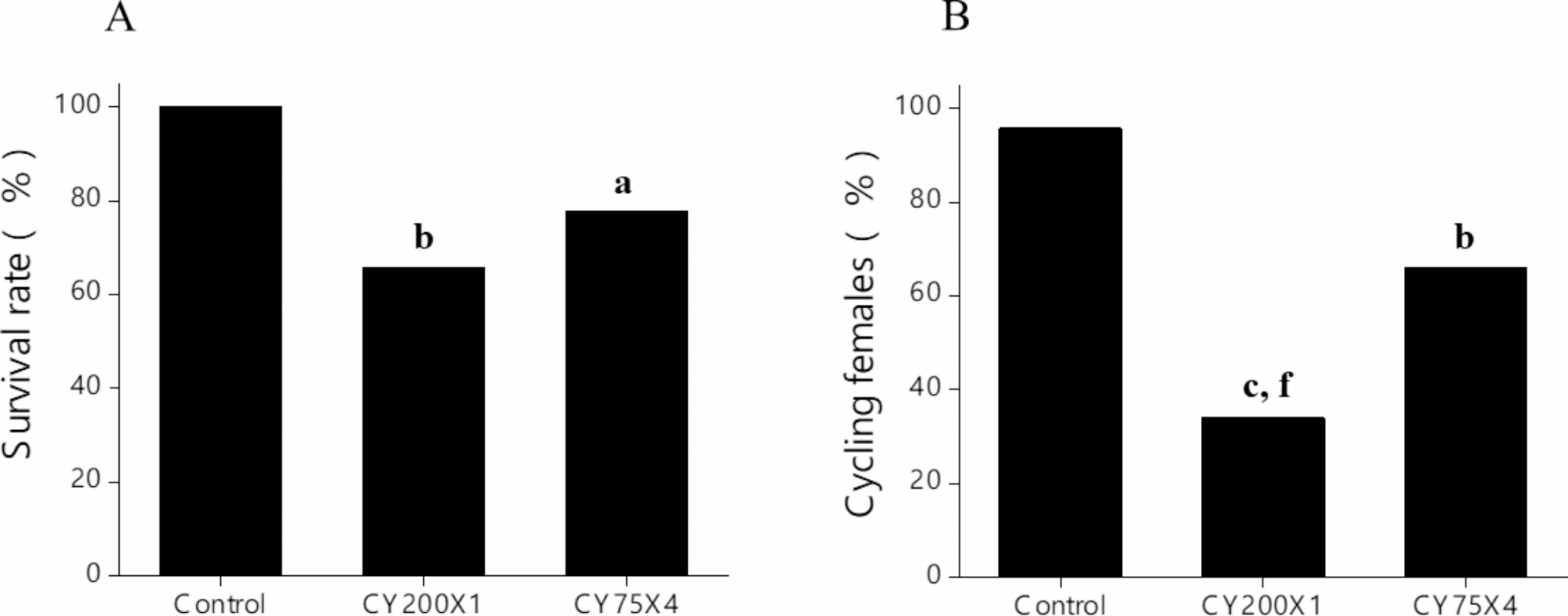
**(A**) Effect of prepubertal exposure to large single (CY200X1) (*N* = 82), small multiple (CY75X4) (*N* = 68) doses and unexposed control (*N* = 24) on survival rate ^**a**^*p* < 0.05, ^**b**^*p* < 0.01 vs. control (**B**) Cycling females at 14-week; females exhibiting estrus stage (solid portion) in CY200X1 (*N* = 82), CY75X4 (*N* = 68), control (*N* = 24). ^**b**^*p* < 0.01, ^**c**^*p* < 0.0001 vs. control; ^**f**^*p* < 0.001 vs. CY75X4.

A significant reduction in body weight was observed in CY200X1 and CY75X4 groups in comparison to the control (*p* < 0.05 − 0.001) until the 13th week of life (Supplementary Table S1). However, CY75X4 group had significantly higher body weight (28.7 ± 0.5 g) than CY200X1 (22.7 ± 0.6 g; *p* < 0.001). The survival rates in both CY200X1 (54/82, 65.8%) and CY75X4 (53/68, 77.9%) were significantly lower than the control group (24/24, 100%; *p* < 0.5) (Fig. 1A).

A two-week-long vaginal cytology assessment at 12–14 weeks revealed a significantly low number of cycling females in CY200X1 (28/82, 34.1%; *p* < 0.0001) and CY75X4 (45/68, 66.2%; *p* < 0.01) groups compared to the control group (23/24, 95.8%; Fig. 1B). Importantly, CY75X4 females had a significantly higher proportion of cycling females than CY200X1 (*p* < 0.001).

### Reduced follicle loss in CY75X4-exposed females

Ovarian weights of cycling females of both CY200X1 (3.3 ± 0.8 g) and CY75X4 (3.9 ± 0.5 g) were comparable to control (3.9 ± 0.5 g). Non-cycling females of CY200X1 showed a significant reduction in ovarian weight (1.5 ± 0.3 g; *p* < 0.05) compared to the control, whereas in CY75X4, the ovarian weights were comparable to the control.

**Fig. 2:**
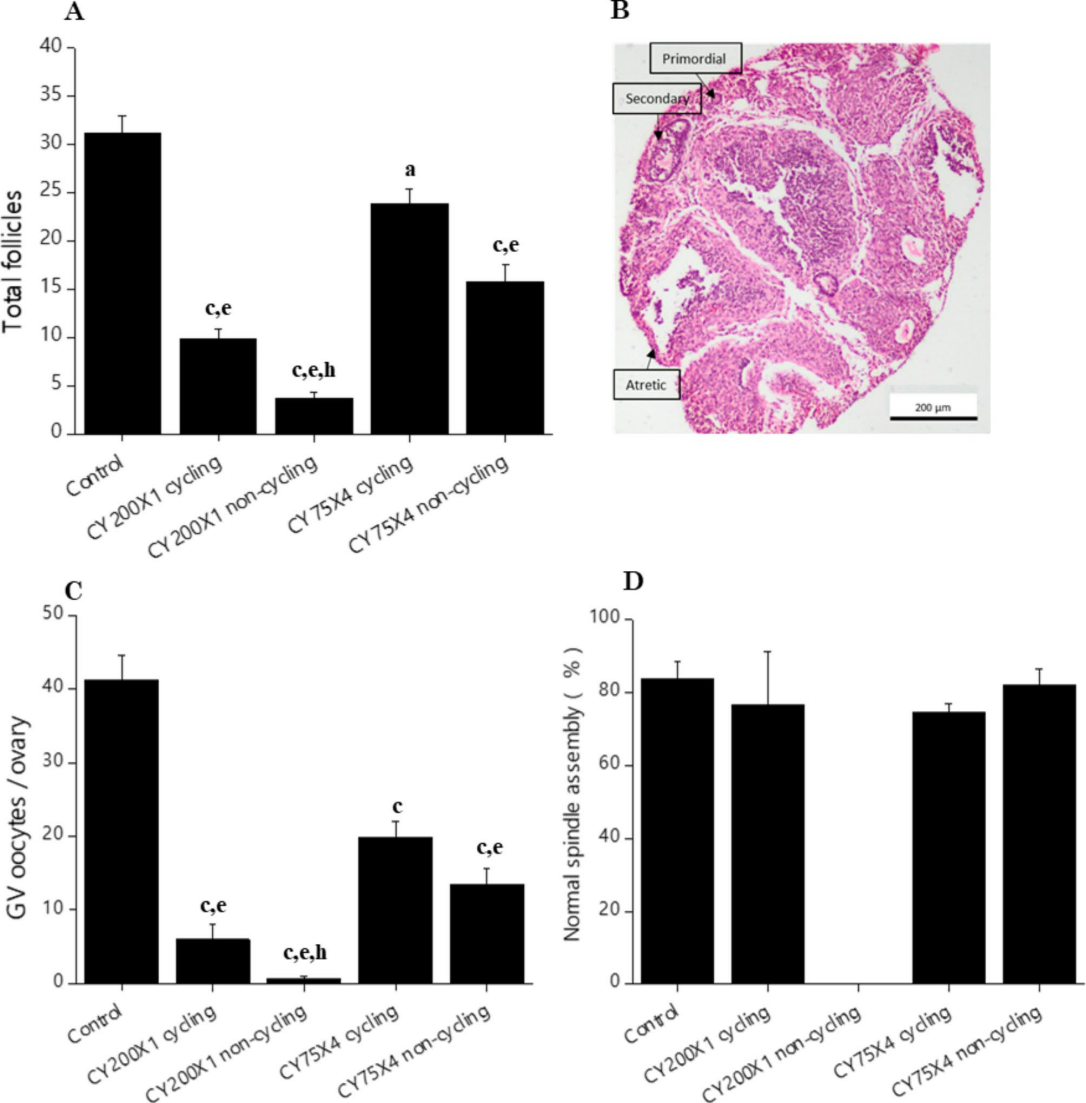
(**A**) Effect of prepubertal exposure to large single (CY200X1) (*N* = 6 cycling & *N* = 6 non-cycling), small multiple (CY75X4) (*N* = 10 cycling & *N* = 8 non-cycling) CY doses and unexposed control (*N* = 6) on the total number of follicles per ovarian section ^**a**^*p* < 0.05, ^**c**^*p* < 0.001 vs. control; ^**e**^*p* < 0.01 vs. CY75X4 cycling; ^**h**^*p* < 0.01 vs. CY75X4 non-cycling (**B**) A representative image of diminished ovarian reserve in CY200X1 non-cycling female mice showing primordial, secondary and atretic follicle (**C**) The number of immature GV oocytes from CY200X1 (*N* = 6 cycling & *N* = 6 non-cycling), CY75X4 (*N* = 10 cycling & *N* = 8 non-cycling) and unexposed control (*N* = 6) per female ^**c**^*p* < 0.001 vs. control; ^**e**^*p* < 0.01 vs. CY75X4 cycling; ^**h**^*p* < 0.01 vs. CY75X4 non-cycling (**D**) The percentage of in vitro matured oocytes with normal spindle assembly from CY200X1 (*N* = 16 cycling & *N* = 0 non-cycling), CY75X4 (*N* = 43 cycling & *N* = 54 non-cycling) and unexposed control (*N* = 74).

A significant reduction in total follicle number was observed in CY200X1 and CY75X4 groups in comparison to control (*p* < 0.05 − 0.001) at the 14th week of life (Fig. 2A). However, CY75X4 cycling females had significantly higher total follicle count than CY200X1 (*p* < 0.001). The non-cycling females of CY200X1 had the lowest counts of primordial, primary, secondary, and antral follicles and were significantly lower than control and CY75X4 cycling females (*p* < 0.05–*p* < 0.001) (Supplementary Table S2). Furthermore, CY200X1 had a significantly lower number of GV oocytes in their ovaries compared to control and CY75X4 cycling females (*p* < 0.01 – *p* < 0.001) (Fig. 2C).

## Association between follicle loss and oocyte competence

The GV oocytes were assessed for their ability to undergo meiotic maturation in vitro. At 24 h post-culture, 66.7 ± 5.6% GVs in the control group progressed to the metaphase II stage (Table 1). Both cycling and non-cycling females of CY200X1 and CY75X4 groups had comparable in vitro maturation rates as that of control. Also, meiotic spindle assembly was not significantly impaired in any of the groups assessed (Fig. 2D). However, cycling CY75X4 females yielded significantly higher number of in vitro matured metaphase II oocytes (10.9 ± 1.2) compared to non-cycling CY200X1 females (0.3 ± 0.2) (Table 1).

**Table 1:** Effect of prepubertal CY exposure on oocyte in vitro maturation.

Groups	Number of females	GV oocytes retrieved	In vitro maturation assessment, 24 h post incubation (mean ± SE)				
			Degenerated oocytes	GV oocytes	M I oocytes	M II oocytes	Maturation rate (%)
Control	06	247 (41.2 ± 3.4)	16 (2.7 ± 1.3)	20 (3.3 ± 0.7)	51 (8.7 ± 2.7)	160 (26.7 ± 1.6)	66.7 ± 5.6
CY200X1-cycling	06	36 (6.0 ± 2.1)	3 (0.5 ± 0.3)	6 (1.0 ± 0.4)	5 (0.8 ± 0.5)^a^	23 (3.8 ± 1.3)^b^	66.7 ± 6.2
CY200X1-non cycling	06	04 (0.7 ± 0.3)	0 (0.0 ± 0.0)	2 (0.3 ± 0.2)^a, d^	0 (0.0 ± 0.0)^b, d^	2 (0.3 ± 0.2)^c, d^	50.0 ± 28.9
CY75X4- cycling	10	198 (19.8 ± 2.2)	9 (0.9 ± 0.3)	39 (3.9 ± 1.3)	41 (4.1 ± 0.9)	109 (10.9 ± 1.2)	56.1 ± 5.1
CY75X4- non cycling	08	108 (13.5 ± 2.1)	1 (0.1 ± 0.1)	15 (1.9 ± 0.6)	20 (2.5 ± 0.8)	73 (9.1 ± 1.6)	68.6 ± 6.6

^a^*p* < 0.05, ^b^*p* < 0.01, ^c^*p* < 0.001 vs. control; ^d^*p* < 0.05 vs. CY75 × 4- cycling.

**Fig. 3:**
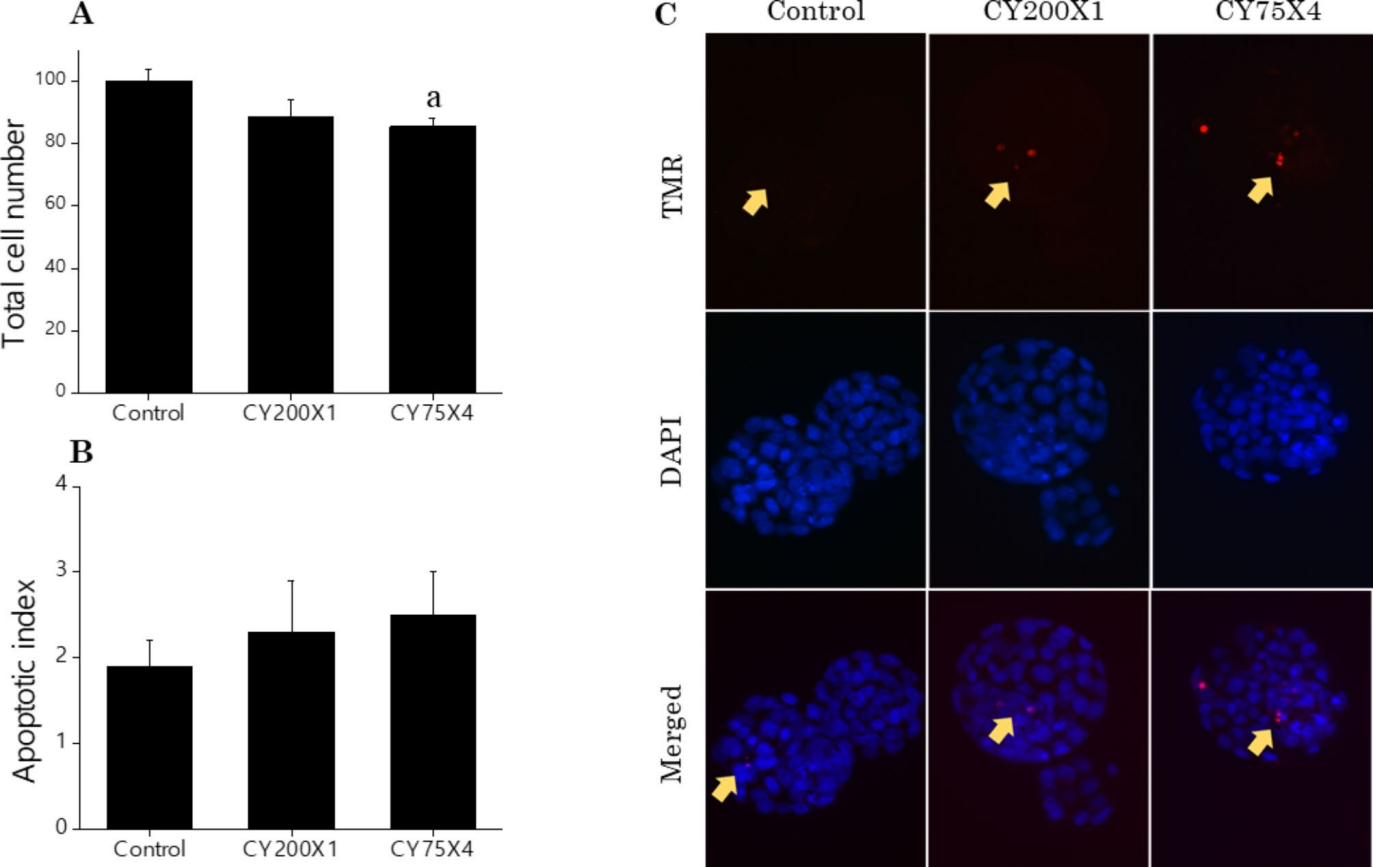
**(A**) Effect of prepubertal exposure to large single (CY200X1) (*N* = 35), small multiple (CY75X4) (*N* = 51) CY doses and unexposed control (*N* = 54) on total cell number ^**a**^*p* < 0.05 vs. control (**B**) Apoptotic index in expanded blastocyst obtained in vitro at 120 hpi (**c**) Representative images of the expanded blastocysts showing TUNEL positive cells.

CY exposure in both experimental groups resulted in the reduction of the number of ovulated oocytes post-superovulation compared to the control (35.3 ± 2.7; *p* < 0.001) (Table 2). Specifically, CY200X1 exposed cycling females had ~ 2-fold lower oocyte yield (11.8 ± 2.1) than CY75X4 exposed cycling females (19.1 ± 1.7; *p* < 0.05). Though fertilization and embryo progression until 72 h post-insemination (hpi) were not affected by CY exposure, blastocyst rates were significantly reduced in CY200X1-exposed cycling females (67.8 ± 4.1; *p* < 0.05) compared to control (85.1 ± 2.4). On the other hand, expanded blastocysts from CY75X4 exposed females had significantly low total cell numbers (88.6 ± 2.6; *p* < 0.05 vs. control; 100.1 ± 3.7) (Fig. 3A), whereas no significant difference in the apoptotic index was observed between any groups (Fig. 3B).

**Table 2:** Effect of prepubertal CY exposure on in vitro fertilization and pre-implantation embryo development.

Groups	Number of females	Oocytes retrieved	MII oocytes inseminated	Embryo progression, in vitro (mean ± SE)			
				2PN, 2 PB at 10 hpi	2-cell at 24 hpi	Morula at 72 hpi	Blastocyst at 120 hpi
Control	06	212 (35.3 ± 2.7)	184 (30.7 ± 1.4)	166 (89.9 ± 3.9)	160 (96.2 ± 2.1)	150 (90.3 ± 2.3)	142 (85.1 ± 2.4)
CY200X1	08	94 (11.8 ± 2.1)^**c, d**^	75 (9.4 ± 1.8)^**c, d**^	61 (86.2 ± 7.5)	52 (88.6 ± 4.2)	48 (78.1 ± 5.5)	43 (67.8 ± 4.1) ^**a**^
CY75X4	09	172 (19.1 ± 1.7)^**c**^	149 (16.6 ± 1.4)^**c**^	133 (87.8 ± 5.1)	124 (94.1 ± 2.7)	108 (81.5 ± 4.2)	100 (74.9 ± 4.2)

^**a**^*p* < 0.05, ^**c**^*p* < 0.001 vs. control; ^**d**^*p* < 0.05 vs. CY75X4.

### Increased postnatal mortality in CY200X1 exposed first generation (F1) pups

**Fig. 4:**
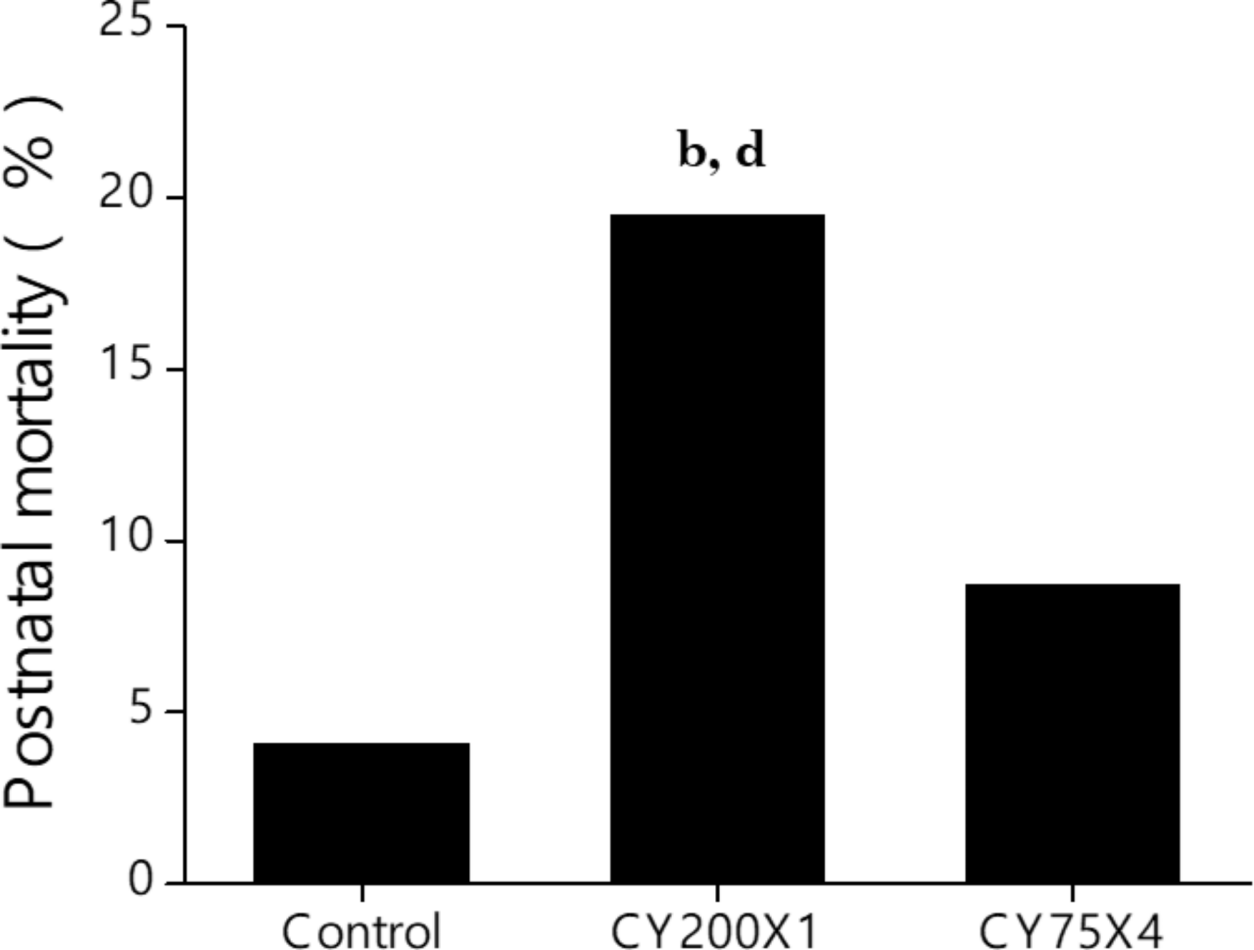
Effect of prepubertal exposure to CY on postnatal mortality of F1 pups in large single (CY200X1) (*N* = 77), small multiple (CY75 × 4) (*N* = 183), and unexposed control (*N* = 74) groups ^**b**^*p* < 0.01 vs. control; ^**d**^*p* < 0.05 vs. CY75X4.

At 14 weeks of life, the mating and fertility index of both CY-exposed groups were found to be similar and comparable to control (Table 3). Also, litter size, litter weight at birth and sex ratio were comparable between the study groups. However, the number of F1 pups that died (15/77, 19.5%) from CY200X1 exposed cycling females was significantly higher than both the control (3/74, 4.1%; *p* < 0.01, Fig. 4) and CY75X4 (16/183, 8.7%; *p* < 0.05) groups.

**Table 3:** Effect of prepubertal CY exposure on mating index and pregnancy outcome.

Groups	Total number of females	Mating index (%)	Fertility index (%)	Litter size (mean ± SE)	Litter weight (mean ± SE) (g)	Male: female	Postnatal mortality (%)
Control	10	9 (90.0)	8 (80.0)	74 (9.3 ± 0.4)	1.6 ± 0.03	1.0	3 (4.1)
CY200X4	14	11 (78.6)	10 (71.4)	77 (7.7 ± 0.7)	1.5 ± 0.03	0.8	15 (19.5) ^b, d^
CY75X4	26	21 (80.8)	20 (76.9)	183 (9.2 ± 0.4)	1.6 ± 0.02	1.2	16 (8.7)

^b^*p* < 0.01 vs. control; ^d^*p* < 0.05 vs. CY75X4.

## Discussion

The current study indicates that the reproductive toxicity exerted on prepubertal ovaries by CY200X1 significantly compromised the ovarian follicle reserve, oocyte competence, and overall fertility potential. On the other hand, the CY75X4 regimen resulted in a smaller fall in follicle number and better fertility outcomes despite a 50% higher total dose of cyclophosphamide. Importantly, the higher postnatal mortality in CY200X1 F1 pups raises concern over the transgenerational risk involved with the large single dose which was not observed in the CY75X4 group.

The human ovary comprises ~ 2 million follicles at birth, which gradually declines due to continuous atresia^12^. Towards the age of 50, the follicular reserve falls below a threshold, resulting in a cessation of the menstrual cycle and culminating in menopause^13^. Chemotherapeutic agents like CY accelerate the rate of follicle loss, affecting reproductive outcomes at a younger age^14^. Beyond a maximum threshold of follicle loss due to toxicological insults, depleted ovarian reserve can cease the estrous cycle^15^. In the present study, the greater follicle loss observed in CY200X1 resulted in a significant population of non-cycling females (31.7%; 26/82) that failed to exhibit the estrus stage and showed a continuous diestrus phase indicating halt in ovulation at early reproductive age (14 weeks). In addition, reduced ovarian weight and ovarian reserve in this group revealed that only one-third of CY200X1 females entered an early reproductive phase of life as cycling females (34.1%; 28/82) as compared to two-thirds of CY75X4 females (66.1%; 45/68).

Studies have shown that CY potentially impairs follicle development and oocyte maturation even long after administration^16^. Assessed 14 weeks after administration, CY200X1 exposure diminished the follicle reserve significantly, with 2.5-fold fewer follicles and GV oocytes compared to the CY75X4 regimen. Additionally, the fewer immature GV oocytes retrieved from the CY200X1 ovaries were competent to undergo maturation and fertilization compared to the control. This indicates that continuous diestrus phase observed in non-cycling females during 12-14th week of age, is not an absolute indicator of depleted ovarian reserve or incompetency of the surviving follicles. Owing to their 2.5-fold higher reserve, CY75X4 cycling females were able to produce 2.5-fold more mature oocytes in vitro, compared to CY200X1 females.

Our earlier study has shown that CY exposure to 2-week-old mice resulted in long-term loss of ovarian reserve without affecting oocyte quality and functional competence^7^. Likewise, oocyte quality, spindle integrity, and fertilizing ability were not affected in both CY75X4 and CY200X1 exposed females. However, a significantly lower blastocyst rate in CY200X1 and impaired blastocyst (expanded) total cell number in CY75X4 at 120 hpi was observed. Aberrant functional behavior of embryos in this study also raises concern about the possibility of delayed DNA damage response and transgenerational risk. P21-mediated checkpoint response is known to delay the cell cycle progression in embryos, specifically at the compaction stage^17^, which could explain reduced cell number in CY75X4 embryos. Though apoptosis functions as a fail-safe strategy to eliminate genetic instability in embryos^17^, there was no significant difference in blastocyst apoptotic index between the two experimental groups. Since gamete-mediated genomic instability can persist in both somatic and germ cell compartments of the first-generation offspring^18^, we speculate that aberrant damage response pathways or increased genomic instability in the fetus resulted in the early post-natal death of the offspring in CY200X1 females. Notably, amongst the 15 deaths in the group, 11 pups died within 24 h, while the other 4 pups died during the weaning period. Importantly, the postnatal mortality in F1 offspring from CY75X4-exposed mothers was significantly lower than CY200X1 group. It is possible that the large single CY dose poses a severe mutagenic or transgenerational risk to F1 pups^8,19^. On the other hand, small multiple CY doses resulted in reduced genetic instability and/or maintained repair process during preimplantation development.

Our findings present a comparative analysis of ovarian reserve and fertility outcomes that help in understanding the dynamics of varying CY dose exposure during the prepubertal period. The implications of large single CY dose versus small multiple doses are three-fold. Firstly, CY200X1 depletes ovarian follicle reserve greatly at exposure, affects the survival of the recipient, and impairs folliculogenesis in the survivors. Secondly, the diminished follicle reserve of CY200X1 yields significantly fewer mature oocytes and/or blastocysts per female that retained ovarian function, and finally, increased postnatal death of F1 pups is a crucial risk for the survivor population. These observations support the claim that the prepubertal period is highly susceptible to a large single CY dose. Hence, small multiple doses of CY could benefit the fertility potential of cancer survivors. This may more accurately reflect the repeated cycles of chemotherapy treatment used in clinical practice. However, we have not explored the relative effects of the two treatment regimens in tumor-bearing animals. Additionally, as CY is not administered alone in cancer treatment, it is essential to explore the behavior of chemotherapeutic agents when combined.

The observations made in this study suggest that small multiple doses of CY can reduce reproductive toxicity in comparison to the large single dose, even with a 50% increase in total dose. This could be attributed to the pharmacokinetics of CY as maximum plasma level of CY metabolites are seen at 2–3 h post-administration^20^. The plasma half-life of CY is approximately 5–12 h and the metabolites are majorly eliminated via urine^21^. In CY200X1, 50% of this large single dose (200 mg/Kg) in active form could be the cause for higher toxicity compared to small doses of 75 mg/Kg. While the present findings highlight the benefit of small multiple doses in protecting long-term fertility and preventing the postnatal mortality of F1 pups, factors such as genomic instability and long-term health consequences in the surviving pups have not been addressed here. In summary, this research has implications for the study of the ovarian toxicity of repeated doses of chemotherapeutic drugs, as used in young prepubertal patients, to model more accurate clinical treatment regimens.

## Materials and methods

### Animals, CY exposure, and follow-up

Swiss albino mice were inbred and housed in the Central Animal Research Facility of the Manipal Academy of Higher Education. Animal handling and maintenance were done in accordance with institutional guidelines for animal experimentation after obtaining prior approval from the Institutional Animal Ethics Committee (Kasturba Medical College, Manipal & Kasturba Hospital Institutional Ethics Committee, approval #IAEC/KMC/73/2020 & #IAEC/KMC/39/2021). The animals were housed and maintained in controlled conditions of 23 ± 2 °C, 12 h light-dark cycle, 50 ± 5% humidity, and were fed with standard diet and water, *ad libitum*.

The study was conducted in accordance with the Animal Research: Reporting of In Vivo Experiment (ARRIVE) guidelines. The experimental design involved a total of one hundred and seventy-four healthy Swiss albino prepubertal female mice, wherein eighty-two mice received a large single dose of 200 mg/Kg cyclophosphamide (C7397, Sigma Aldrich, USA) (hereafter referred to as CY200X1) intra-peritoneally (*i.p.*) at postnatal day 14. A second group of sixty-eight mice received small multiple CY doses of 75 mg/Kg *i.p*. for 4 consecutive weeks at postnatal day 14, 21, 28, and 35 (hereafter referred to as CY75X4) (Supplementary Fig. 1). In parallel, twenty-four healthy mice received four weekly doses of normal saline and served as controls for the study.

CY-exposed mice were monitored for fur loss, drop in body weight gain, and survival rates. At 12 weeks of life, the estrous cycle was monitored daily using vaginal saline flush for two weeks, as described earlier^22^. Females that showed cornified epithelium on vaginal cytology were confirmed to be in the estrus stage and were considered cycling females. Females that showed predominant infiltration of leukocytes continuously over two weeks were considered non-cycling females. Following the estrous cycle observations, the cycling and non-cycling females were assessed at 14 weeks of life for ovarian reserve and oocyte quality. Oocyte competence and fertility index were studied only in cycling females.

### Ovarian reserve and oocyte quality assessment

Animals were sacrificed by cervical dislocation, and their ovaries were extracted and weighed. One of the ovaries from each animal was subjected to histological analysis by Hematoxylin & Eosin (H&E) staining, while the other ovary was completely teased under a stereomicroscope using ultra-fine needles to release germinal vesicle (GV) oocytes from secondary/tertiary follicles. Histological assessment involved only the follicles with a clear oocyte nucleus, and analysis was performed by assessing the total follicle count (Fig. 2B). The number of primordial, primary, secondary and antral follicles was estimated in a 5 μm ovarian section under a light microscope, as discussed earlier^7^. A minimum of 6 ovaries were taken per group.

The GV oocytes were subjected to in vitro maturation (IVM) and spindle analysis as described previously^23^. Briefly, the oocytes were cultured in 20 µL Dulbecco’s Modified Eagle’s Medium (DMEM, D5648 Sigma Aldrich, USA) supplemented with 1% non-essential α-amino acids (M7145 Sigma Aldrich, USA), 1% Insulin-Transferrin-Selenium (51500-056 Gibco, India), 10 µM pyruvate (P3662 Sigma Aldrich, USA), and 0.3% bovine serum albumin (MB083 Sigma Aldrich, USA), overlaid with oil, and incubated at 37 °C, 5% CO_2_ for 24 h. Maturation was confirmed by the extrusion of the first polar body.

Metaphase-II oocytes were permeabilized in an extraction buffer (1 h at 37 °C) and fixed in ice-cold methanol (15 min at − 20 °C). Blocking was done using 5% knockout serum (10828-010 Gibco, India) in 0.25% triton X-100 (1 h at 37 °C). Oocytes were incubated overnight at 4 °C in primary anti-α-tubulin antibody (1:150, T9026 Sigma Aldrich, USA) followed by treatment with FITC tagged goat anti-mouse IgG antibody (1:500, NB7535 Novus Biologicals, USA) for 1 h at 37 °C. The chromosomes were stained with 4 µg/mL DAPI (40,6-Diamidino-2-phenylindole D9542 Sigma Aldrich, USA) and observed under the fluorescent microscope (Imager-A1, Zeiss, Gottingen, Germany). The spindle images were captured under 40X objective lens using LAS X software (Leica microsystems, Germany).

### Superovulation, in vitro fertilization, and embryo assessment

Females were superovulated using 5 IU of pregnant mare serum gonadotropin (PMSG, Sigma Aldrich, USA) followed by 10 IU of human chorionic gonadotropin (hCG, Eutrig-HP) after 48 h. Cumulus-oocyte-complexes (COCs) were collected from the oviduct after 13 h and subjected to i*n vitro* fertilization and embryo culture as described earlier^7^. Embryo development was monitored until the blastocyst stage at regular intervals. The expanded blastocysts were collected at 120 hpi for quality assessment and were fixed in 4% paraformaldehyde for 1 h. Terminal deoxynucleotidyl transferase (TdT) dUTP Nick End Labelling (TUNEL) assay was performed as described earlier^24^ using TUNEL reaction mixture (12156792910, Roche Diagnostics, USA). Apoptotic index was calculated using the formula, total apoptotic cells / total cell number x 100, per blastocyst.

### Fertility index and pregnancy outcome

Cycling females at the proestrus stage were housed with healthy, proven fertile male mice at a 1:1 ratio overnight for mating at 14 weeks of life. Successful mating was confirmed by the presence of a vaginal plug. Mating efficiency (mating index) was calculated using the formula: females with plug/females mated x 100. Females with plug were housed individually until parturition. Fertility index was calculated using the formula: total number of deliveries / total number of females mated x 100. These data are represented as percentages.

At parturition, litter size, litter weight, and sex ratio were noted. The litter was observed during the weaning period till 4 weeks of age: cumulative postnatal deaths were recorded and represented as a postnatal mortality rate.

### Statistical analysis

Data were analyzed using one-way analysis of variance (ANOVA) if normally distributed and by the Kruskal-Wallis test if not normally distributed, followed by Dunn’s post-hoc test. Categorical data were analyzed using the Chi-square test. Data were represented as mean ± SEM. All tests were performed using the GraphPad InStat 3.0 statistical package (GraphPad Inc., USA) and all the graphs were plotted using Origin 8.0 (Origin Lab Corporation, Northampton, MA, USA).

## Electronic supplementary material

Below is the link to the electronic supplementary material.

Supplementary Material 1

Supplementary Material 2

Supplementary Material 3

## Data Availability

All data generated or analyzed during this study are included in this published article and its supplementary information files.

## Declarations

### Approval for animal experiments

The study was approved by the Institutional Animal Ethics Committee (Kasturba Medical College, Manipal & Kasturba Hospital Institutional Ethics Committee) approval# IAEC/KMC/73/2020 & IAEC/KMC/39/2021).
