# Supplementary figures and images for: Comparison of large single and small multiple doses of cyclophosphamide exposure in mice during early prepubertal age on fertility outcome
Source: Sci Rep. 2024 Dec 28;14:31042. doi: 10.1038/s41598-024-82264-3 (PMC11681079; doi:10.1038/s41598-024-82264-3)

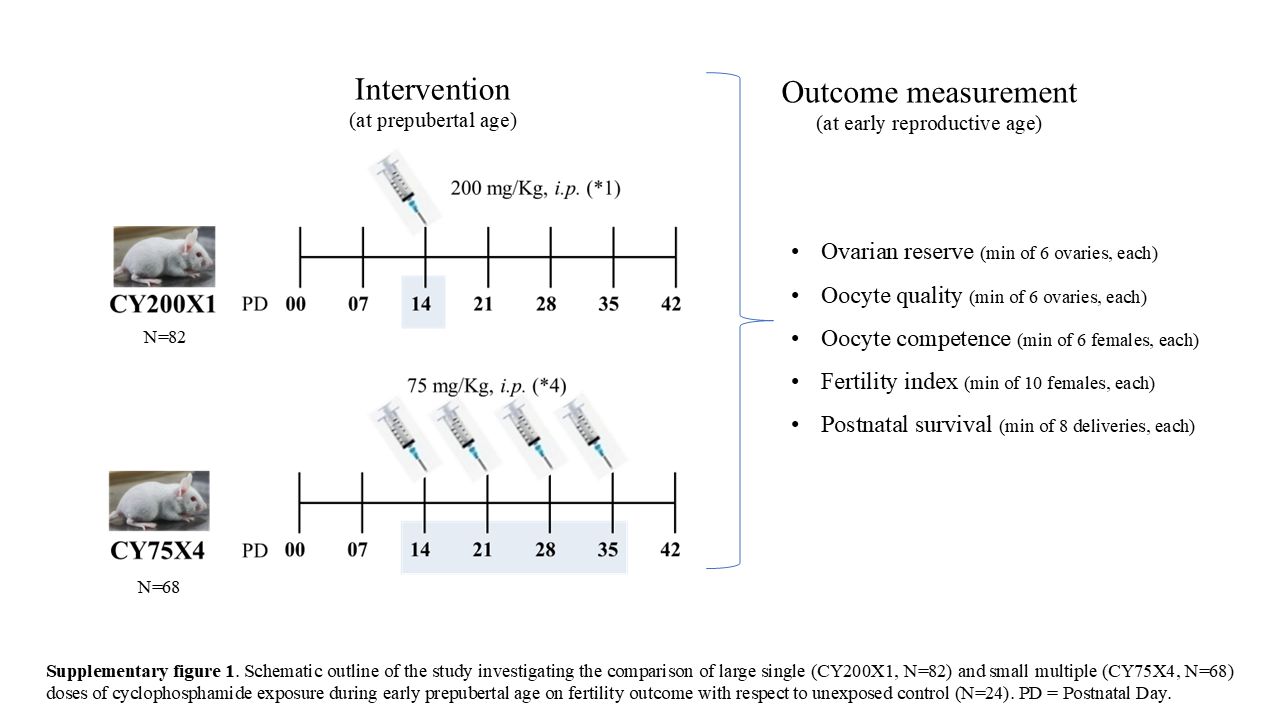

Supplement: Supplementary file 1 — Supplementary Material 1 [file 41598_2024_82264_MOESM1_ESM.tif]
