# Supplementary material for: Comparison of large single and small multiple doses of cyclophosphamide exposure in mice during early prepubertal age on fertility outcome
Source: Sci Rep. 2024 Dec 28;14:31042. doi: 10.1038/s41598-024-82264-3 (PMC11681079; doi:10.1038/s41598-024-82264-3)
Supplement: Supplementary file 2 — Supplementary Material 2 [file 41598_2024_82264_MOESM2_ESM.docx]

Supplementary Table S1: Effect of prepubertal CY exposure on body weight of Swiss albino female mice

| Groups | Number of females | Body weight (g) measurement at varying age (week) | | | | | |
| --- | --- | --- | --- | --- | --- | --- | --- |
|  |  | 2 | 3 | 4 | 5 | 9 | 13 |
| Control | 24 | 8.6 ± 0.2 | 13.9 ± 0.3 | 20.3 ± 0.7 | 25.5 ± 0.8 | 32.1 ± 1.0 | 35.2 ± 1.0 |
| CY200X1 | 82 | 8.4 ± 0.1▀ | 9.1 ± 0.2 ^c, f^ | 12.6 ± 0.3 ^c, f^ | 16.9 ± 0.4 ^c, f^ | 17.7 ± 0.4 ^c, f^ | 22.7 ± 0.6 ^c, f^ |
| CY75X4 | 68 | 8.5 ± 0.2 * | 11.6 ± 0.2 *^b^ | 16.9 ± 0.4 *^a^ | 21.2 ± 0.4 *^b^ | 26.5 ± 0.6 ^a^ | 28.7 ± 0.5 ^c^ |

Data is presented in mean ± SE

Time of CY administration

▀ Large single CY dose of 200 mg/Kg body weight, i.p.

*Small multiple CY doses of 75 mg/Kg body weight, i.p.

^a^p < 0.05, ^b^p < 0.01, ^c^p < 0.001 *vs*. control; ^f^p < 0.001 *vs*. CY75X4
