# Supplementary material for: Comparison of large single and small multiple doses of cyclophosphamide exposure in mice during early prepubertal age on fertility outcome
Source: Sci Rep. 2024 Dec 28;14:31042. doi: 10.1038/s41598-024-82264-3 (PMC11681079; doi:10.1038/s41598-024-82264-3)
Supplement: Supplementary file 3 — Supplementary Material 3 [file 41598_2024_82264_MOESM3_ESM.docx]

Supplementary Table S2: Effect of prepubertal CY exposure on number of follicles in the ovarian reserve at 14 weeks of life

| Groups | Number of ovaries | Primordial  follicle | Primary  follicle | Secondary  follicle | Antral  follicle | Total  follicles |
| --- | --- | --- | --- | --- | --- | --- |
| Control | 6 | 56 (9.3 ± 1.2) | 27 (4.5 ± 0.8) | 68 (11.3 ± 0.7) | 37 (6.2 ± 0.7) | 187 (31.2 ± 1.8) |
| CY200X1 cycling | 6 | 11 (1.8 ± 0.7) **^b^** | 24 (4.0 ± 0.7) | 15 (2.5 ± 0.5) **^b,d^** | 9 (1.5 ± 0.7) **^a^** | 59 (9.8 ± 1.1) **^c,f^** |
| CY200X1 non cycling | 6 | 7 (1.2 ± 0.5) **^c,d^** | 6 (1.0 ± 0.3) **^b,d^** | 6 (1.0 ± 0.3) **^c,f^** | 3 (0.5 ± 0.2) **^c,e^** | 22 (3.7 ± 0.6) **^c,f,h^** |
| CY75X4 cycling | 10 | 60 (6.0 ± 0.9) | 37 (3.7 ± 0.5) | 99 (9.9 ± 0.9) | 43 (4.3 ± 0.5) | 239 (23.9 ± 1.5) **^a^** |
| CY75X4 non cycling | 8 | 43 (5.4 ± 1.1) | 19 (2.4 ± 0.3) | 49 (6.1 ± 0.7) | 16 (2.0 ± 0.5) **^a^** | 126 (15.8 ± 1.7) **^c,d^** |

Data is presented in mean ± SE.

**^a^**p < 0.05, **^b^**p < 0.01, **^c^**p < 0.001 *vs.* control; **^d^**p < 0.05, **^e^**p < 0.01, **^f^**p < 0.001 *vs* CY75X4 cycling; **^h^**p < 0.01 *vs* CY75X4 non-cycling
